# Development of a cationic polyethyleneimine-poly(lactic-*co*-glycolic acid) nanoparticle system for enhanced intracellular delivery of biologics†

**DOI:** 10.1039/d3ra06050k

**Published:** 2023-11-17

**Authors:** Shannon R. Tracey, Peter Smyth, Una M. Herron, James F. Burrows, Andrew J. Porter, Caroline J. Barelle, Christopher J. Scott

**Affiliations:** a Queen's University Belfast, The Patrick G Johnston Centre for Cancer Research 97 Lisburn Road Belfast Northern Ireland BT9 7AE UK; b Queen's University Belfast, School of Pharmacy Belfast BT9 7BL UK; c Elasmogen Aberdeen UK

## Abstract

Intracellular delivery of proteins, peptides and biologics is an emerging field which has the potential to provide novel opportunities to target intracellular proteins, previously deemed ‘undruggable’. However, the delivery of proteins intracellularly remains a challenge. Here, we present a cationic nanoparticle delivery system for enhanced cellular delivery of proteins through use of a polyethyleneimine and poly-(lactic-*co*-glycolic acid) polymer blend. Cationic nanoparticles were shown to provide increased cellular uptake compared to anionic and neutral nanoparticles, successfully delivering Variable New Antigen Receptors (vNARs), entrapped within the nanoparticle core, to the cell interior. vNARs were identified as ideal candidates for nanoparticle entrapment due to their remarkable stability. The optimised 10% PEI-PLGA nanoparticle formulation displayed low toxicity, was uniform in size and possessed appropriate cationic charge to limit cellular toxicity, whilst being capable of escaping the endo/lysosomal system and delivering their cargo to the cytosol. This work demonstrates the ability of cationic nanoparticles to facilitate intracellular delivery of vNARs, novel biologic agents with potential utility towards intracellular targets.

## Abstract 2

Cationic PEI-PLGA nanoparticles are shown as a superior delivery platform, displaying enhanced intracellular delivery of biologics.

## Graphical abstract

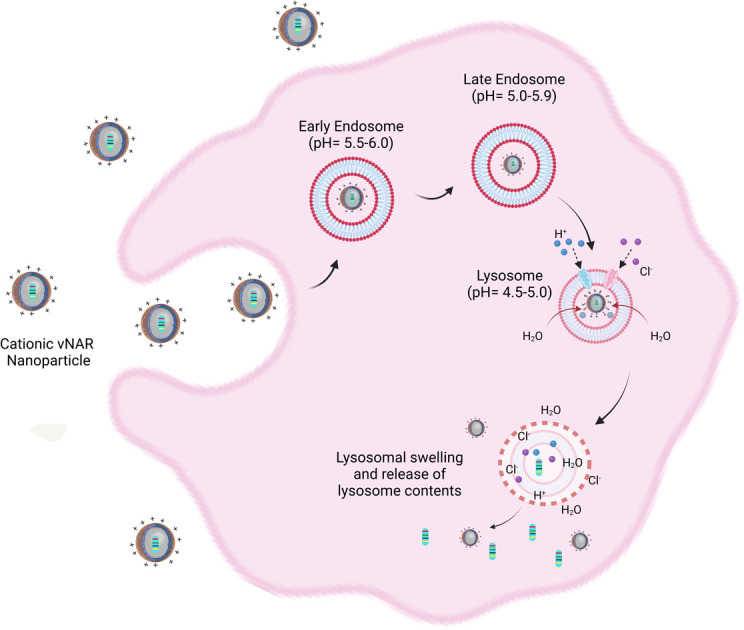

## Introduction

Completion of the human genome project almost 20 years ago led to the identification of a number of novel prospective drug targets, particularly within the field of cancer.^1,2^ A vast majority of these targets are intracellular and although several have been identified as prevalent tumour drivers, many remain ‘undruggable’, largely due to a lack of a suitable binding pocket, as well as sub-optimal therapeutic delivery by conventional approaches. Clinically approved drugs can be divided into two broad classes; small molecules, and biologics (which include monoclonal antibodies, proteins, peptides, and vaccines).^3^ Whilst small molecules have been successfully employed as targeted cancer therapies, they have been shown to be ineffective against a range of intracellular proteins, mostly due to their inability to block the large surface area of the target protein and/or impede protein–protein interactions due to their hydrophobic nature. As a result, interest has grown in utilising protein-based therapeutics in this setting. Following advancements in the design process and manufacturability, protein therapeutics have becoming increasingly fruitful, comprising ∼50% of the top ten best selling drugs globally in 2020.^4,5^

To date, a large portion of protein delivery approaches have been effectively directed towards extracellular targets, as exemplified by a range of monoclonal antibodies.^6^ However, intracellular delivery has proven challenging due to the intrinsic properties of proteins, such as their high molecular weight, as well as an intrinsic polarity which prevents them from being transported across the cell membrane. Various approaches have been investigated in efforts to deliver proteins intracellularly such as physical/mechanical methods (*e.g.*, microinjection and electroporation) and covalent protein modification including the integration of cell penetrating peptides. Whilst showing promise, these methods are invasive and have been shown to elicit some level of inherent cellular toxicity.^7–9^ As an alternative approach, the use of nanocarriers has sparked significant interest for the intracellular delivery of therapeutic agents, such as small molecules, peptides, mRNA, DNA, and larger proteins. Nanocarriers represent a versatile formulation platform enabling development of nanoparticles with a breadth of size and surface functionalisation, in turn facilitating enhanced cellular uptake, prolonged circulation times as well as active cell targeting.^10,11^

Cationic polymeric nanoparticles exhibit attractive characteristics for the delivery of biologics intracellularly; namely enhanced cellular uptake as a result of electrostatic interactions between cationic nanoparticles and the anionic cell membrane proteins and an ability to facilitate endosomal escape whilst preserving entrapped protein integrity.^12–15^

In this study, we wish to examine the capabilities of a cationic nanoparticle formulation for the intracellular delivery of proteins, through the development of a PEI-PLGA based nanoparticle entrapping Variable New Antigen Receptors (vNARs) as a model protein for enhanced intracellular delivery. vNARs possess many unique properties attributed from their distinctive origin. Other than their small size and high affinity for their target, the inherent stability of vNARs make them ideal candidates for the harsh conditions within the cell.^16,17^ Furthermore, the presence of disulfide bridges, results in vNARs having a protruding structure, which in turn leaves the structure of vNARs predisposed to binding inside deep fissures, where they have the ability to bind into deep cryptic epitopes which would usually be concealed to conventional antibodies and other proteins.^17–20^ As a result of exposure to harsh conditions within in their natural environment, of the shark sera, vNARs have adapted to their environment resulting in a molecule which possesses considerable stability. vNARs have been shown to be stable over a range of external conditions, including changes in temperature, pH, and the presence of proteases.^16,18–21^ Following synthesis and determination of the physical characteristics of the vNAR PEI-PLGA nanoparticle system, the enhanced ability of the cationic nanoparticle to escape the endo–lysosomal system was assessed using *in vitro* cell based assays. Overall, this study identifies a potential platform through which the well-established limitations of biologics in targeting intracellular proteins may be overcome.

## Materials and methods

All chemicals were purchased from Sigma-Aldrich and were >97.0% purity unless stated otherwise.

### Experimental

#### vNAR phage display library screening

vNARs were isolated from a Elasmogen's proprietary synthetic vNAR libraries using phage display panning techniques as described previously.^22,23^

#### PLGA-PEI nanoparticle formulation

PEI-PLGA nanoparticles were prepared by a double emulsion solvent evaporation method producing 10 mg of nanoparticles per batch. Resomer® RG 502H – poly(d,l-lactide-*co*-glycolide) acid terminated, (lactide : glycolide 50 : 50; *M*_w_ 7000–17 000 Da) (PLGA) was dissolved DCM, where a 10–25% (w/v) of polyethyleneimine (PEI) was added to the organic phase resulting in a PEI-PLGA polymer blend, resulting in 10 mg of polymer in 2 mL DCM. For example, for the final 10% PEI-PLGA formulation, nanoparticles were prepared by dissolving 9 mg Resomer® RG502H PLGA in 1.8 mL of DCM, where 200 μL of 5 mg mL^−1^ PEI in DCM was added to the organic phase, resulting in a 10% w/v PEI-PLGA polymer blend. The organic phase was injected dropwise into the aqueous phase consisting of 0.5 mL of 1% PVA in 50 mM MES hydrate buffer (pH 5). While stirring at 1000 rpm (multichannel stirrer, model MS-53M, Lab Companion) on ice. Emulsification was achieved by sonication in pulse mode (3 seconds on, followed by two seconds off) for 36 seconds at an amplitude of 50% using a Model FB120 sonic dismembrator (Fisher Scientific). 10 mL of 1% PVA/MES buffer solution was then added and emulsified by sonication in pulse mode (3 seconds on, followed by two seconds off) for 54 seconds at an amplitude of 50%. Vials were left stirring at 1000 rpm overnight to ensure evaporation of the organic solvent. The formed nanoparticles were purified *via* centrifugation (17 000×*g*, at 4 °C, 20 min) using PBS, by three wash–spin cycles. Nanoparticles were adjusted to a concentration of 10 mg polymer per mL in PBS and stored at 4 °C until further usage. Alternatively, non-cationic, control PLGA nanoparticles were formulated. Where necessary, vNAR entrapped nanoparticles were formed using the method described, with the addition of 10 μg of D8 FITC tagged vNAR (unless otherwise stated) added per mg polymer to the initial aqueous of the nanoparticle formulation.

#### Nanoparticle characterisation

Nanoparticle diameter and polydispersity (PDI) were measured *via* dynamic light scattering (DLS) using a Nanobrook Omni instrument (Brookhaven Instruments Corporation, NY, USA). All nanoparticle samples were evaluated following resuspension at 100 μg polymer per mL in 2 mL of 0.05% PBS (v/v) in dH_2_O. Zeta potential was measured *via* laser doppler micro-electrophoresis. All measurements were recorded in triplicate, with results expressed as mean ± SEM. Nanoparticle size and dispersity was also determined by nanoparticle tracking analysis (NTA) using the NanoSight NS300 (Malvern Instruments, UK), with nanoparticles were prepared at 200 μg polymer per mL in dH_2_O.

#### Environmental scanning electron microscopy

Nanoparticles were resuspended at 5 mg polymer per mL in dH_2_O. Double-sided copper tape was fixed to aluminium stubs and 5 μL of the nanoparticle solutions applied dropwise and allowed to dry. Nanoparticles were then sputter-coated with gold and imaged using a Quanta 250 FEG ESEM (FEI) at ×30 000 magnification.

#### Nanoparticle stability studies

For stability studies, nanoparticles were stored at room temperature (RT) or 4 °C in suspension at 1 mg polymer per mL in 1× PBS. Samples were pelleted by centrifugation and resuspended before assessing characteristics. Alternatively, 1 mg polymer pellets were stored at −20 °C with the isolated pellet resuspended in 1 mL of dH_2_O prior to analysis. Stability study samples were assessed in terms of particle diameter, PDI, zeta potential using the Nanobrook Omni (Brookhaven) instrument.

#### Western blotting

Cells were collected and lysed in radio immunoprecipitation (RIPA) buffer (10 mM Tris, 150 mM NaCl, 1 mM Na_2_EDTA·2H_2_O, 1% Triton X-100, 0.1% SDS supplemented with protease inhibitor cocktail (Millipore)) and lysates collected. Protein concentrations were determined, and 200 μg of total protein per sample was taken forward for his-Ni NTA pulldown. Pull down samples were collected and denatured for analysis by SDS-PAGE and transferred using a BioRad system. Membrane was blocked with 5% milk powder in 0.1% PBST under agitation for 1 h at RT. The membrane was incubated in primary antibody (anti-6X his – ab18184) at 4 °C overnight with agitation before incubation with an anti-mouse-HRP conjugated secondary antibody for 1 h at room temperature. Proteins were detected using ECL substrate – ultra highly sensitive and imaged using a G: BOX Chemi XX6 gel doc system (Syngene) and GeneSys software.

#### Assessment of cell viability

CellTiter-Glo® luminescent cell viability assay (Promega G7570) was used to determine nanoparticle toxicity. Following the treatment period, spent media was aspirated and assay conducted according to manufacturer's instructions luminescence was read using a Biotek Synergy HT plate reader and cell viability expressed relative to untreated control cells (treated with 1× PBS).

#### Immunofluorescence

HeLa cells were seeded in 8-well chamber glass culture slides (BD Falcon) and treated with relevant nanoformulations, in duplicate, at a concentration of 200 μg polymer per mL for 45 minutes at 37 °C with 5% CO_2_. Post-treatment, cells were gently washed with ice-cold PBS (×3) and treated with acid strip buffer (50 mM glycine, 150 mM NaCl in PBS, pH 3) to remove non-internalised nanoparticles. Cells were fixed with 4% w/v paraformaldehyde and permeabilized using 0.5% v/v Triton X-100 in PBS. After fixation and permeabilization, cells were blocked overnight at 4 °C in blocking buffer (10% Goat Serum v/v and 1% w/v BSA in PBS) before incubating with the relevant primary antibody: goat anti-mouse anti-lysosomal-associated membrane protein 1 (LAMP1) antibody (Abcam (ab25630); 1 : 50 dilution in blocking buffer) or goat anti-rabbit EEA1 antibody (cell signalling technology (c45b10); 1 : 50 dilution in blocking buffer). Following this, incubation with goat anti-mouse IgG secondary antibody Alexafluor568 (1 : 1000 dilution in blocking buffer, ThermoFisher Scientific (A-11031) for LAMP-1) or goat anti-rabbit IgG secondary antibody Alexafluor568 (1 : 1000 dilution in blocking buffer, ThermoFisher Scientific (A-1103) for EEA1). Cells were washed ×3 with ice cold 1× PBS. Coverslips were mounted onto the glass slides using Vectashield antifade mounting medium with DAPI (Vector Laboratories, H-1200) and sealed before imaging using confocal microscopy.

#### Confocal microscopy imaging

Imaging was performed using Leica SP8 confocal microscope, and Leica LAS-X software. Fluorescent images were attained post excitation with a UV emitting diode (405 nm) and argon (488 nm); DPSS (561 nm); or HeNe (543 nm, 594 nm, and 633 nm) lasers as required. Images were taken at 1 or 2× zoom using a HCX PL APO 1.4-0.6NA 63x oil immersion objective with a 1024 × 1024 frame, 12 bit-depth and 600 Hz scan speed. Images presented per experimental series were acquired using standardized settings and parameters. Image analysis was conducted using Leica LAS X software.

#### Cationic nanoparticle induced endo/lysosomal destabilisation

The ability of cationic 10% PEI-PLGA nanoparticles to escape the endo/lysosomal compartment was assessed *via* two methods: calcein localization by confocal microscopy and acridine orange staining by flow cytometry. For calcein localization studies, HeLa cells were treated with calcein (Life Technologies) (2 mg mL^−1^ in cell culture media) ± (0.2 mg mL^−1^ PLGA or 10% PEI-PLGA nanoparticles in PBS buffer) for 3 h. Cells were washed before fixing, permeabilization and addition of coverslip as above. For acridine orange endo/lysosomal integrity assays, HeLa cells were treated, as detailed (0.02–0.5 mg mL^−1^), for 24 and 48 h. On completion of treatment, cells were washed with PBS (×2), trypsinized and pelleted by centrifugation. Cells were then resuspended in PBS buffer containing 1 μg mL^−1^ acridine orange (Life Technologies) and incubated for 15 min at 37 °C. Sample analysis was then conducted by flow cytometry (BD Accuri C6 Plus Flow Cytometer).

#### Statistical analysis

Statistical tests were performed using GraphPad Prism software (version 9.1.2) and employed as detailed in each figure legend. Statistical significance is indicated by asterisks on graphs, where level of significance is defined in corresponding figure legends. The degree of colocalisation was quantified *via* Pearson's correlation coefficient (*R*) using ImageJ colocalisation analysis, where *R* +1 indicated perfect association and *R* < 0, a negative association, and no correlation.

## Results

### Preparation and characterisation of PEI cationic nanoparticles

To facilitate formulation of a cationic nanoparticle, a polymer blend of PLGA and PEI was chosen. PLGA was selected as the bulk polymer due to its slow release, as well as its FDA ‘generally regarded as safe’ status.^24,25^ Polyethyleneimine (PEI) is a branched polycationic polymer which has been extensively used for delivery of proteins and nucleic acids into living cells.^26,27^ This is as a result of its high positive charge density and chain flexibility when compared to other polycations.^27–29^ PEI-PLGA nanoparticles were prepared using various percentage polymer blends (10–25%), with PEI content represented as a percentage of the total polymer content. Increasing the percentage of PEI polymer added in the organic phase resulted in nanoparticles which displayed a greater cationic charge (+6.01 mV ± 3.86 – +14.44 mV ± 7.79), when compared to PLGA nanoparticles (−11.07 mV ± 0.98) (Table 1). Not only was there a change to nanoparticle charge when the percentage of PEI was increased, an increase in nanoparticle size was also observed, with blank PLGA nanoparticle diameter of 232.98 nm ± 9.07 increasing to a diameter of 250–260 nm for PEI nanoparticle formulations. This was associated with a marginal increase in PDI (Table 1).

**Table 1:** Physicochemical properties of nanoformulations. Physicochemical properties of PLGA nanoparticles (NP) and PEI-PLGA nanoparticles were assessed *via* DLS. Nanoparticles were resuspended at 100 μg mL^−1^ in 0.05% (v/v) PBS in dH_2_O and assessed by DLS in terms of particle diameter, polydispersity, and zeta potential. Data presented as mean ± SEM (*n* = 3)

Nanoparticle	Particle diameter (nm)	PDI	Zeta potential (mV)
PLGA NP	232.98 ± 9.07	0.103 ± 0.015	−11.07 ± 0.98
10% PEI-PLGA NP	250.29 ± 1.45	0.114 ± 0.001	+6.01 ± 3.86
15% PEI-PLGA NP	260.28 ± 16.80	0.142 ± 0.005	+8.04 ± 4.08
20% PEI-PLGA NP	261.28 ± 11.07	0.142 ± 0.003	+11.65 ± 1.40
25% PEI-PLGA NP	259.02 ± 13.55	0.149 ± 0.020	+14.44 ± 7.79

**Fig. 1:**
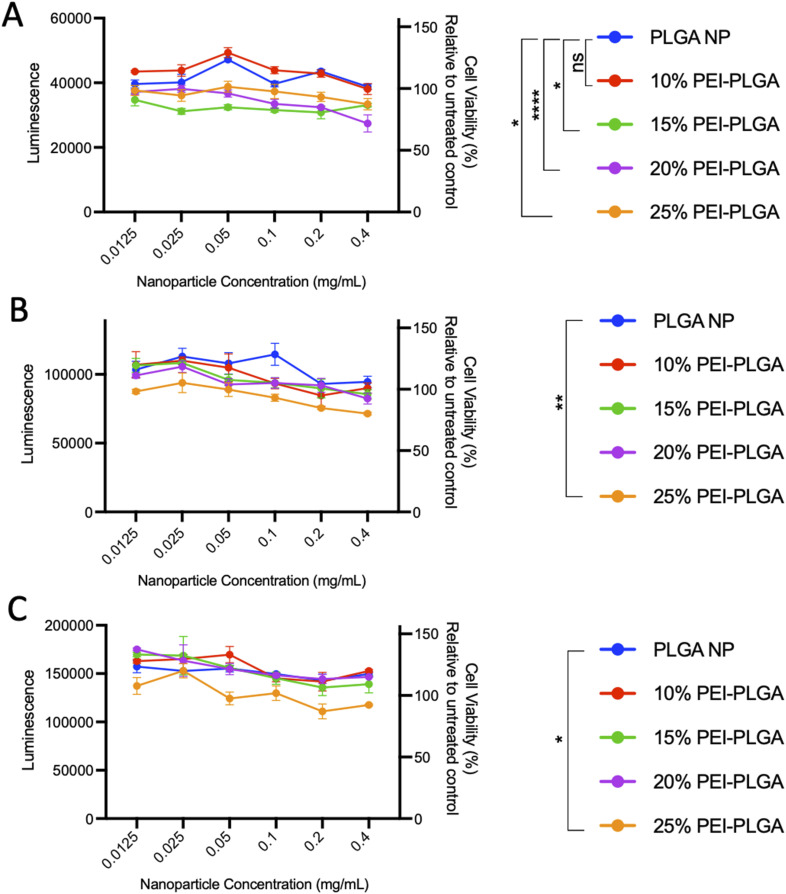
Evaluation of PEI-PLGA nanoparticle formulation. To determine cell viability, HeLa cells were treated with 0.012–0.4 mg per mL of PLGA or 10–25% PEI-PLGA nanoformulations. Cell viability was assessed (A) 24, (B) 48 and (C) 72 h post-treatment using CTG assay (Promega). Assay preformed in triplicate; data expressed as mean ± SEM. Statistical significance determined using a two-way ANOVA and Dunnett's multiple comparison's test (ns or no line denotes no significance, *p < 0.1, **p < 0.01, ***p < 0.001, ****p < 0.0001) where significance is denoted in legend.

In order to evaluate nanoparticle toxicity, each formulation was tested *in vitro*. For the purposes of this study, HeLa cells were selected as a relevant cell model for this proof-of-concept study due to their ease of use in ability to analyse cellular toxicity and applicability for confocal microscopy with nanoparticle uptake/localisation studies. HeLa cells were treated with varied concentrations of the formulations (0.012–0.4 mg per mL polymer) for up to 72 h and cell viability assessed. At 24 h post-treatment, cells treated with 20% and 25% PEI-PLGA nanoparticles showed a small but significant decrease in cell viability at nanoparticle concentrations >0.1 mg mL^−1^ (Fig. 1A). At both 48- and 72 h timepoints, a decrease in cell viability was observed with 15–25% PEI-PLGA nanoformulations as polymer concentration increased (Fig. 1B and C), whereas 10% PEI-PLGA nanoparticles showed little to no toxicity at the highest assessed concentration; comparable to the PLGA nanoparticle control. Therefore, the 10% PEI-PLGA nanoformulation was selected for all further studies.

**Fig. 2:**
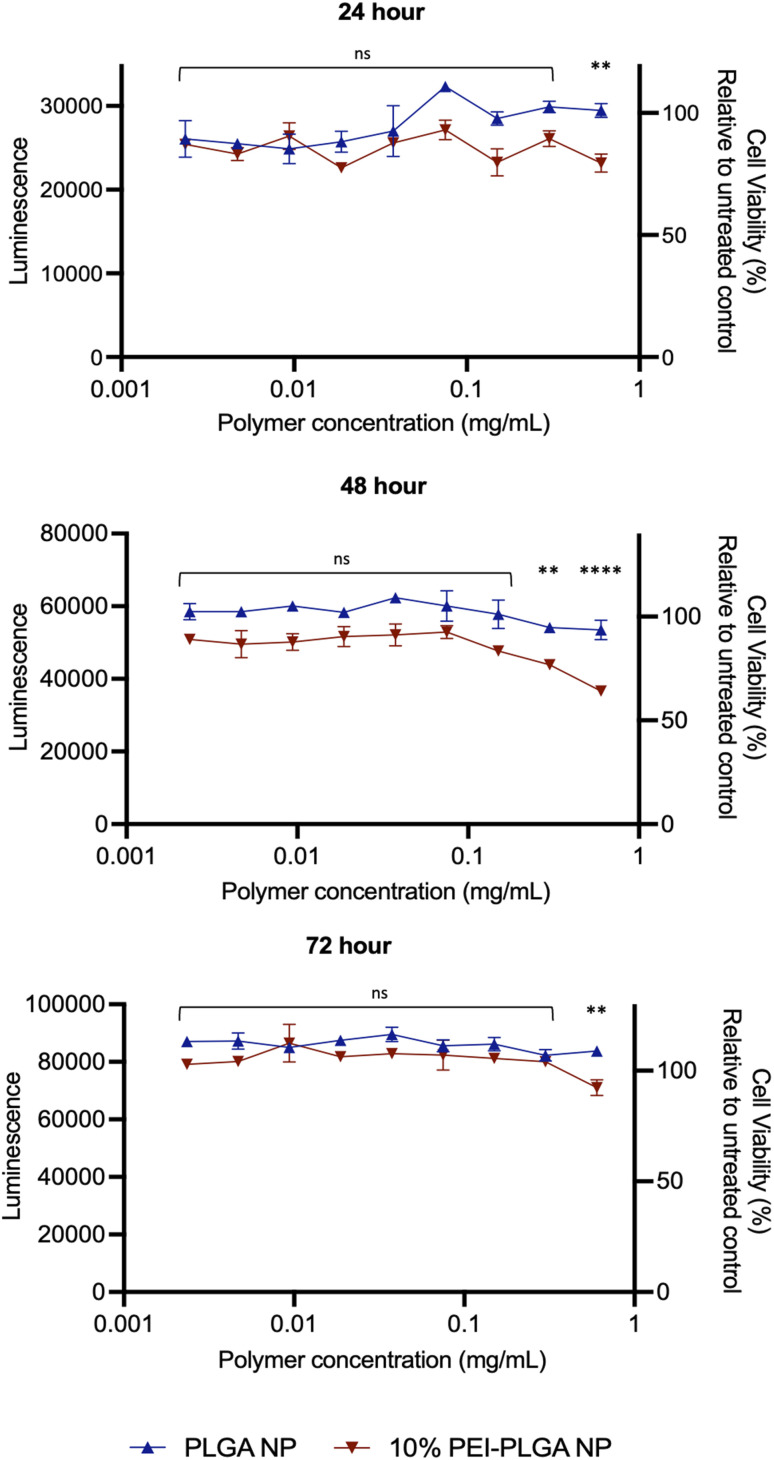
Cationic nanoparticles show minimal decrease in cell viability compared to anionic control. To determine cell viability, HeLa cells were treated with 0.003–0.6 mg per mL of PLGA or 10% PEI-PLGA nanoparticles (NP). Cell viability was assessed 24, 48 and 72 h post-treatment using CTG assay (Promega). Assay performed in triplicate; data expressed as mean ± SEM. Representative of three independent experiments. Statistical significance determined using a two-way ANOVA (ns denotes no significance, **p < 0.01, ****p < 0.0001) where significance is denoted in legend.

Cytotoxicity of the final 10% PEI-PLGA nanoparticle formulation was further assessed *via* Cell Titer- Glo® (CTG), by measuring cell viability in HeLa cells after 72 h treatment. The incubation of positively charged 10% PEI-PLGA nanoparticles lead to a significant reduction cell viability in HeLa cells, particularly after 48- and 72 h treatment at the highest concentration (0.6 mg per mL polymer), compared to the PLGA nanoparticle control (Fig. 2). However, at polymer concentrations <0.5 mg mL^−1^ nanoparticles show a negligible decrease in cell viability. Next, the stability of each nanoformulation was assessed, where both PLGA and 10% PEI-PLGA nanoparticles were subjected to a range of storage conditions (+4 °C, −20 °C and RT). For a period of up to 21 days, the physical characteristics of each nanoparticle formulation (diameter, PDI and zeta potential) was assessed. The PLGA and 10% PEI-PLGA nanoparticles were both found to have a diameter of ∼250 nm, where no significant change was observed in the baseline characteristics of both PLGA and 10% PEI-PLGA nanoparticles, whilst PDI remained <0.2, demonstrating a monodisperse nanoparticle suspension and the zeta potential for each formulation remained relatively consistent over the time course of the study at all three storage conditions (ESI 2†).

### Assessment of endo–lysosomal disruption

**Fig. 3:**
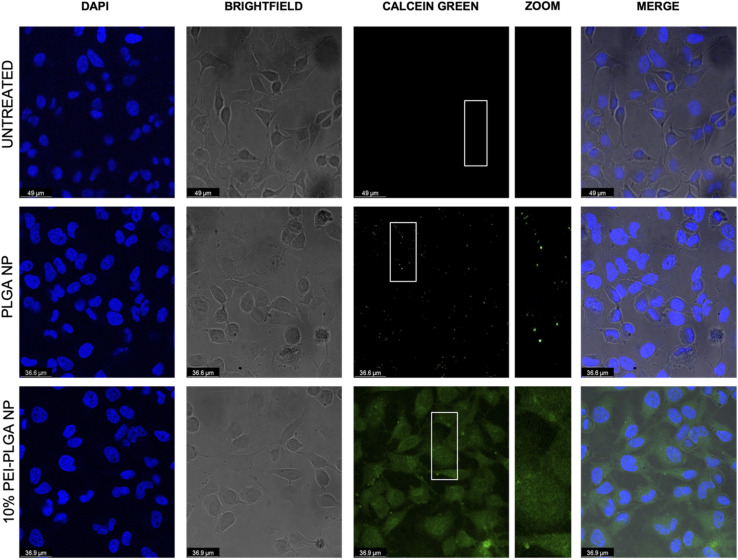
Assessment of endo/lysosomal disruption following nanoparticle treatment using calcein. HeLa cells were treated simultaneously with calcein (2 mg mL^−1^) and PLGA nanoparticles, 10% PEI-PLGA nanoparticles (0.2 mg mL^−1^) or PBS (untreated control). Following 3 h of treatment cells were fixed, permeabilised and stained with DAPI for visualisation of nuclear regions. Slides were imaged using confocal microscopy with DAPI (blue), calcein (green) and merged images shown. Images representative of three independent experiments.

The behaviour of the nanoparticles once internalised and ability to escape the endo–lysosomal system was next studied. Firstly, calcein tracking confocal microscopy and acridine orange staining *via* flow cytometry was employed to assess the ability of the cationic nanoparticle formulation to enhance nanoparticle escape form the endo/lysosomal pathway. Calcein is a cell impermeable, fluorescent dye, which has previously been utilised to assess endosomal membrane stability.^30,31^ As calcein is cell impermeable, internalisation results in staining of the endosomal lumen following endocytosis. Cells incubated with calcein alone (untreated), showed no staining indicating there was no internalisation of the dye alone, without nanoparticle uptake (Fig. 3). Whereas those cells incubated with the anionic PLGA nanoparticle (charge −11.07 mV ± 0.98) in combination with calcein displayed punctate staining, which is indicative of the presence of the dye within the endocytic vesicles, showing that the calcein has been up taken alongside the PLGA nanoparticles *via* endocytosis resulting in staining of the endosomal lumen. The staining pattern observed is very clear punctate staining, highlighting vesicle integrity has been preserved, and nanoparticles are within the endosome (Fig. 3). Upon destabilisation and disruption of the endo–lysosomal membrane, this results in membrane leakage, thus resulting in leaching of calcein from the endosome. As a result, the calcein dye is released into the wider cytosolic region, appearing as a dispersed cytosolic staining pattern. This is observed following treatment with the cationic 10% PEI-PLGA nanoparticle formulation (charge +6.01 mV ± 3.86), where staining is observed as being more diffuse throughout the cytosolic space, in stark contrast to the aforementioned punctate staining seen with control PLGA nanoparticles (Fig. 3). This signifies that the 10% PEI-PLGA nanoparticles are capable of disrupting the endosomal membrane sufficiently to escape the endo–lysosomal pathway.

Endo–lysosomal escape was further confirmed *via* an acridine orange assay. HeLa cells were treated with 0.02–0.5 mg mL^−1^ of both PLGA and 10% PEI-PLGA nanoparticles for 24 and 48 h. Following treatment, cells were collected and stained by incubating with acridine orange. Acridine orange is a fluorescent dye which accumulates within acidic regions, such as the lysosome. Following lysosomal disruption, the fluorescent signal of acridine orange diminishes and becomes more diffuse, suggesting the dye is being released within the more neutral environment of the cytosol, essentially escaping the lysosome.^32^

**Fig. 4:**
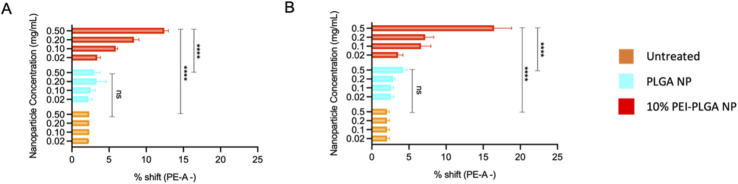
Cationic nanoparticles are capable of causing endo/lysosome disruption. The ability of cationic nanoparticles to escape the lysosome was assessed *via* acridine orange staining. HeLa cells were treated with 0.02–0.5 mg mL^−1^ of PLGA or 10% PEI-PLGA nanoparticles. Following treatment cells were stained with acridine orange and cell fluorescence detected *via* flow cytometry. Data shown as % shift of PE-cells (shift to the left), where disruption in observed in a concentration dependant manner following (A) 24 h and (B) 48 h relative to untreated control. Data expressed as mean ± SEM (*n* = 3). Statistical significance was established by one-way ANOVA and Tukey's *post hoc* test (ns or no line denotes no significance and ****p < 0.0001).

This diminished staining profile is observed by a loss of fluorescence (a shift to the left, (ESI 3†)) when compared to the untreated control. Following treatment of HeLa cells with 10% PEI-PLGA nanoparticles, the acridine orange signal is significantly reduced in comparison to PLGA nanoparticles and untreated cells. Indeed, fluorescent signal was seen to be concentration dependant, with respect to the 10% PEI-PLGA nanoparticles at both 24 and 48 h time points (Fig. 4). This highlights again the ability of the cationic nanoparticle formulation to escape the endo–lysosomal system and subsequently delivery their cargo to the cytosol.

### Entrapment of a hydrophilic vNAR within nanoparticle core

**Fig. 5:**
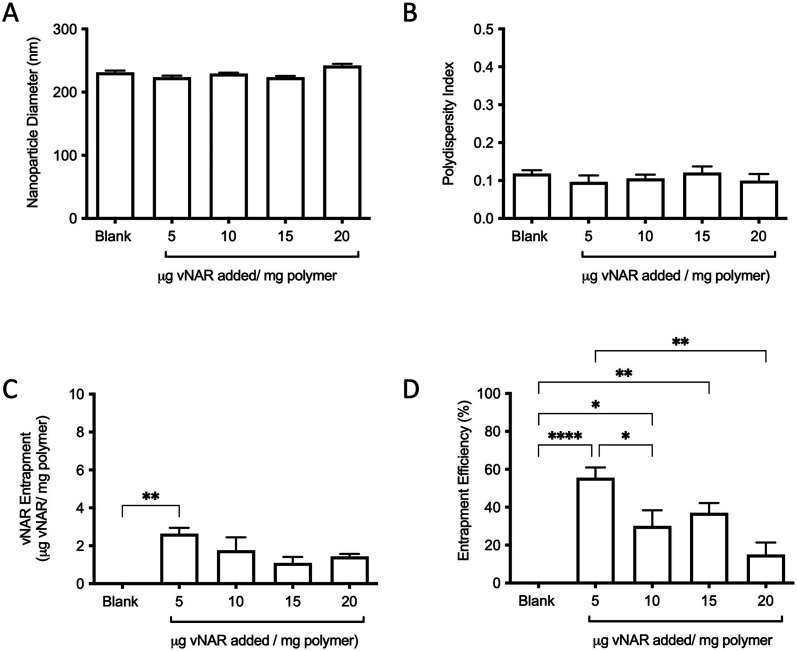
Characterisation of vNAR entrapment within nanoparticle core. Various amounts of fluorescent D8-FITC vNAR (5–20 μg per mg polymer) were added during nanoparticle synthesis, with physicochemical properties of blank PLGA nanoparticles and vNAR nanoparticles subsequently assessed by DLS in terms of (A) particle diameter, and (B) polydispersity index. (C) vNAR entrapment and entrapment efficiency (D) were assessed through measurement of fluorescence and extrapolated from a standard curve. Measurements were conducted in triplicate and presented as mean ± SEM. Statistical significance determined using one-way ANOVA and Tukey's *post hoc* test (ns or no line denotes no significance, *p < 0.1, **p < 0.01, ***p < 0.001, ****p < 0.0001).

For the purpose of these studies, a model vNAR was selected, containing a FITC tag to accommodate quantification of vNAR entrapment, as well as downstream localisation studies. In the first instance, the ability to entrap the vNAR within the nanoparticle core was assessed using the PLGA nanoparticle control, where varied quantities of vNAR (5–20 μg per mg polymer) were added to the aqueous phase during nanoparticle synthesis. After washing, vNAR loaded nanoparticles (subsequently referred to as vNAR nanoparticles) were assessed to determine physiochemical properties and vNAR entrapment. vNAR nanoparticles displayed diameters (∼200 nm) comparable to the PLGA nanoparticle control (Fig. 5A), whilst all formulations demonstrated a PDI <0.2, indicating a monodisperse nanoparticle suspension. vNAR entrapment was assessed following addition of between 5-20 μg vNAR per mg polymer. Interestingly, it was observed that there was no clear correlation of vNAR concentration in the formulation input and resultant entrapment, with entrapment efficiency demonstrating a reducing trend with increased vNAR concentration (Fig. 5C and D). Optimal vNAR entrapment was observed with 5 μg vNAR added to the formulation mix (1.70 ± 0.98 μg per mg polymer), with the optimal entrapment efficacy (Fig. 5D). Given that increasing amounts of vNAR input did not lead to enhanced entrapment (Fig. 5C), this formulation was selected for further studies.

**Fig. 6:**
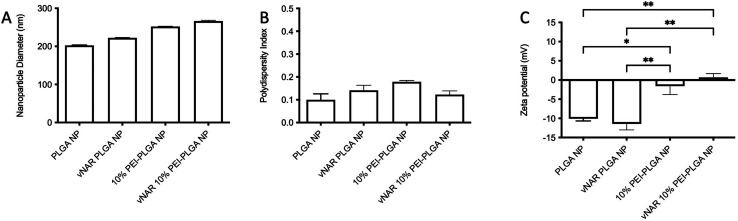
Characterisation of 10% PEI-PLGA NP formulation. Physicochemical properties of PLGA nanoparticles and 10% PEI-PLGA vNAR entrapped nanoparticles were assessed *via* DLS in terms of (A) particle diameter and (B) polydispersity index. (C) Zeta potential was measured *via* phase analysis light scattering (PALS). Nanoparticles were resuspended at 100 μg mL^−1^ in 0.05% PBS (v/v) in dH_2_O for analysis. Measurements conducted in triplicate and presented as mean ± SEM (*n* = 3). Statistical significance determined the using one-way ANOVA and Tukey's multiple comparisons test (ns or no line denotes no significance, *p < 0.1, **p < 0.01).

Following formulation of both 10% PEI-PLGA cationic and vNAR entrapped nanoformulations, both formulation parameters were combined to obtain a cationic nanoparticle delivery system for enhanced delivery of entrapped cargo, in this instance, vNARs. vNAR loaded 10% PEI-PLGA nanoparticles were synthesised as above, and physiochemical characteristics assessed *via* DLS (Fig. 6), and SEM (ESI 4A–D†). Nanoparticle diameter was in anticipated range (∼200–250 nm), with a negligible increase in nanoparticle diameter observed for vNAR loaded nanoparticles (Fig. 6). Importantly, vNAR 10% PEI-PLGA nanoparticles displayed similar vNAR entrapment to that of the vNAR PLGA nanoparticles (Table 2) where an increase in cationic nanoparticle charge observed with the PEI nanoformulation. Nanoparticle size and dispersity were further assessed, with nanoparticles resuspended in dH_2_O (0.1 mg mL^−1^) and analysed *via* NTA. NTA measurements were equivalent to trends obtained by DLS and further emphasised the monodispersity of these systems (ESI 4E†).

**Table 2:** Characterisation of vNAR entrapped nanoparticles. Nanoparticle (NP) diameter was assessed *via* DLS. vNAR entrapment and entrapment efficiency was assessed *via* measurement of fluorescence and extrapolated from a standard curve (*n* = 3)

	Average diameter (nm)	vNAR entrapment (μg per mg polymer)	Entrapment efficiency (%)
vNAR PLGA NP	252.50 ± 1.05	2.63 ± 0.30	52.66 ± 6.16
vNAR 10% PEI-PLGA NP	249.26 ± 2.13	2.80 ± 0.23	56.00 ± 4.62

Nanoparticle stability is a critical aspect of formulation development, as it can affect nanoparticle biodistribution resulting in unwarranted systemic toxicity.^33^ Therefore, stability of the nanoparticle systems was assessed. Nanoparticle physical characteristics, such as size, PDI, zeta potential and vNAR entrapment were assessed over a period of up to 10 days (ESI 5†). Throughout the course of this study, all nanoparticles synthesised were of ∼250 nm in size, with a slight size increase observed for those which were loaded with vNAR. There were no significant changes observed in the baseline characteristics of all nanoformulations assessed throughout the duration of study, demonstrating the stability of both cationic nanoparticle charge and vNAR entrapment in formulations.

### vNAR retains binding affinity for target following nanoparticle formulation

**Fig. 7:**
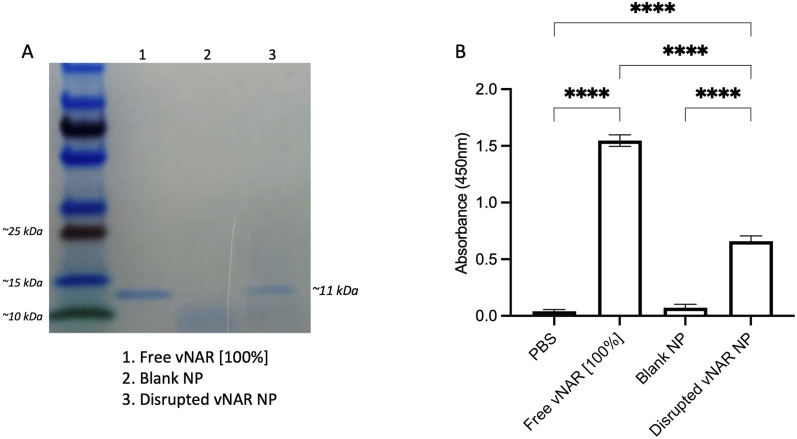
vNAR retains binding affinity for its target antigen following release from nanoparticle. 10% PEI-PLGA nanoparticles were physically disrupted *via* incubation at 95 °C for 2 h followed by intense sonication in an attempt to release entrapped vNAR. The supernatant was collected, and polymer pellet discarded. (A) Supernatant were prepared for analysis by SDS-PAGE. Lane (1) free vNAR (indicative of 100% nanoparticle entrapment) (2) blank nanoparticle supernatant (3) disrupted vNAR nanoparticle supernatant. The bands observed from the supernatant released from the disrupted vNAR nanoparticle appear to be of the same size of the free vNAR (∼11 kDa). (B) Binding of vNAR to its cognate antigen was assessed *via* ELISA, where free vNAR (indicative of 100% nanoparticle entrapment) was used as a control compared to supernatants collected from disrupted nanoparticles. Data presented as mean ± SEM (*n* = 3). Statistical significance determined using one-way ANOVA and Tukey's *post hoc* test (ns or no line denotes no significance, ****p < 0.0001).

Although it has been found that vNARs exhibit remarkable stability, such as temperature range and fluctuations in pH,^16^ their ability to withstand the harsh sonication conditions and solvents of nanoparticle synthesis is unknown. Therefore, following confirmation of successful vNAR entrapment, it was important to evaluate vNAR activity following the nanoparticle formulation process. vNAR loaded 10% PLGA-PEI nanoparticles were physically disrupted and supernatant collected for assessment *via* SDS-PAGE. The bands observed from the supernatant released from the nanoparticle appear to all be of the same size (∼11 kDa), indicating that the vNAR remains intact following release, where as expected, bands appear weaker than that of the free vNAR band (equivalent to 100% entrapment) (Fig. 7A). The ability of vNARs to refold and retain binding activity in a range of harsh biological conditions is a key attribute to their use for nanoformulation and intracellular delivery, therefore in addition to confirmation of vNAR release from nanoparticles, it is important to assess whether the vNAR can retain its functional activity after escaping the nanoparticle and is not somehow impaired within the nanoparticle synthesis process. In order to assess the vNAR binding, nanoparticles were disrupted, and supernatants collected as above. An ELISA was then performed in order to assess binding of disrupted vNAR nanoparticle supernatants to its cognate antigen (not disclosed) using anti-his HRP detection of the vNAR C-terminal his-tag. An increase in relative binding response was observed for the vNAR disrupted nanoparticle, in comparison to blank PLGA nanoparticle control binding (Fig. 7B). This suggests that despite the solvents and sonication techniques used within nanoparticle synthesis, the vNAR retains its binding activity. Despite there being a significant increase in binding observed between free vNAR and the disrupted vNAR nanoparticle, this is likely due to the case of residual vNAR still retained within the particle due to the mild nanoparticle disruption approach employed. Moreover, a significant increase in binding is observed with the vNAR disrupted nanoparticle in comparison to PBS and blank nanoparticle control, indicating that any vNAR released from the particle is still functional and retains its binding activity for its cognitive antigen, despite the reduction in binding observed compared to the free vNAR, which is likely due to not all vNAR being released from the nanoparticle.

### Cationic nanoparticles exhibit enhanced cellular uptake

**Fig. 8:**
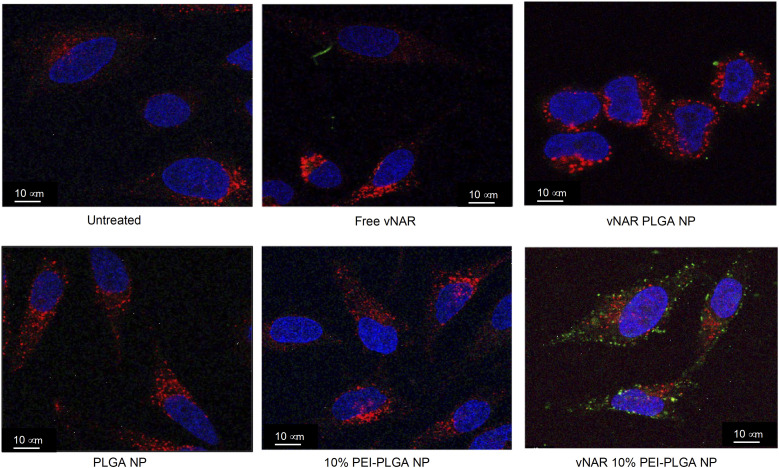
10% PEI-PLGA nanoparticles exhibit enhanced cellular uptake in comparison to PLGA nanoparticles. Nanoparticle internalisation was assessed in HeLa cells by confocal microscopy. Cells were seed at 50 000 cells per well in an 8-well chamber slide and left overnight to adhere. Cells were treated with 0.2 mg mL^−1^ of various nanoparticles (PLGA, 10% PEI-PLGA, vNAR PLGA and vNAR 10% PEI-PLGA) and relevant controls for 1 h. Post treatment, cells were washed with both PBS and an acid strip wash before fixing cells for permeabilization and staining. Lysosomal regions were stained using anti-LAMP1 antibody (red) and nuclear regions stained (blue) using DAPI. FITC conjugated vNAR entrapped within the nanoparticle core can be visualised in green. Images representative of three independent experiments. Scale bars = 10 μm.

**Fig. 9:**
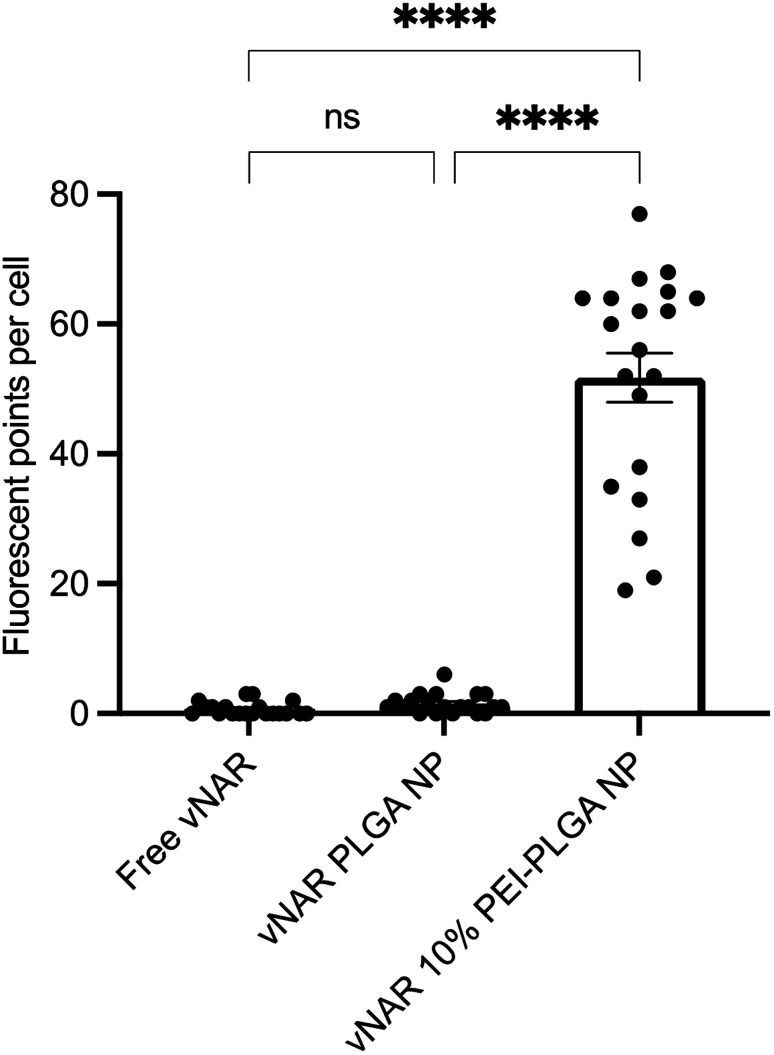
Quantification of nanoparticle uptake in cells. Nanoparticle uptake between treatment groups (free vNAR, vNAR PLGA nanoparticles and vNAR 10% PEI-PLGA nanoparticles) was quantified by determining the number of fluorescent points per cell per individual experiment. Each treatment was conducted in duplicate (2 wells), where 20 cells were selected at random for counting per treatment group. Experiment performed in triplicate, with data presented as mean ± SEM (*n* = 3). Statistical significance was established by one-way ANOVA and Tukey's *post hoc* test (ns or no line denotes no significance and ****p < 0.0001).

Cationic nanoparticles are thought to exhibit a greater internalisation rate in comparison to neutral or anionic nanoparticles, owing to the overall net negative charge of the plasma membrane resulting in the electrostatic attraction of cationic nanoparticles.^34,35^ Thus, the effect of cationic nanoparticles on cellular uptake was evaluated following treatment of HeLa cells with vNAR loaded PLGA and 10% PEI-PLGA nanoformulations. Cells were treated with 200 μg per mL of vNAR 10% PEI-PLGA nanoparticles and vNAR PLGA control nanoparticles, in order to assess nanoparticle uptake in cells. Cells were imaged using confocal microscopy (Fig. 8), where nanoparticle uptake was quantified by assessing number of fluorescent points per cell, representative of FITC tagged vNAR localisation within the cell (Fig. 9).

### Cellular localisation studies with nanoparticle formulations

**Fig. 10:**
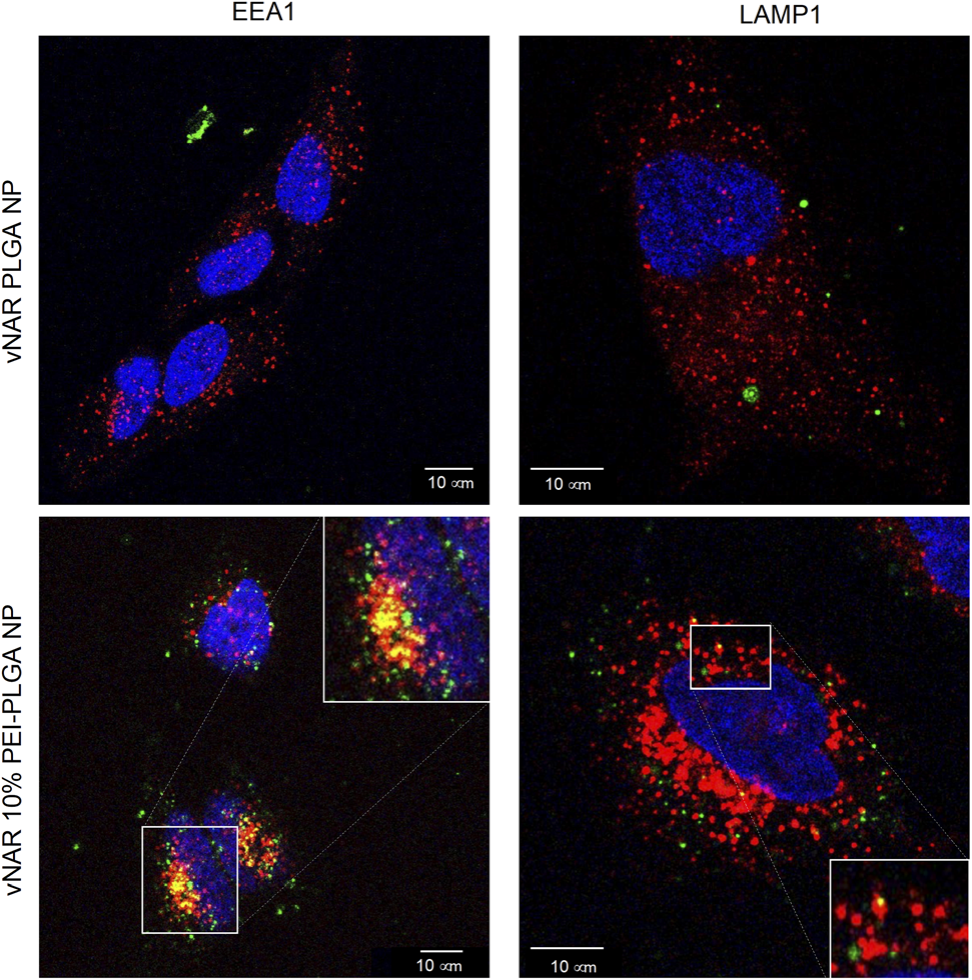
Assessment of nanoparticle localisation within the cell. Localisation of nanoparticles following treatment was assessed in HeLa cells using confocal microscopy. Cells were seeded at 50 000 cells per well in an 8-well chamber slide and left overnight to adhere. Cells were treated with 0.2 mg mL^−1^ of either vNAR PLGA or vNAR 10% PEI-PLGA nanoparticles for 1 h. Post treatment, cells were washed with both PBS and an acid strip wash before fixing cells for permeabilization and staining. Lysosomal and endosomal regions were stained using anti-LAMP1/anti-EEA1 antibody respectively, visualised in red. Nuclear regions were stained using DAPI, visualised in blue. FITC conjugated vNAR entrapped within the nanoparticle core can be visualised in green. Images representative of three independent experiments. Scale bars = 10 μm.

To determine the subcellular localisation of both nanoformulations after internalisation, cells were treated with each nanoformulation as above, and subsequently stained with organelle-specific fluorescent markers, including DAPI, EEA1 and LAMP1 in order to visualise nuclei, early endosomes and lysosomes respectively. Merged images detail that not only is there a significant increase in cationic vNAR 10% PEI-PLGA nanoparticle uptake as previously observed, but after 1 h of treatment, vNAR 10% PEI-PLGA nanoparticles are present within the endosome, denoted by the colocalisation of staining with EEA1 (Fig. 10). Little to no colocalization of nanoparticles was observed with LAMP1 within the lysosome (after timepoints of 30 min–4 h (images shown for 1 h timepoint).

**Fig. 11:**
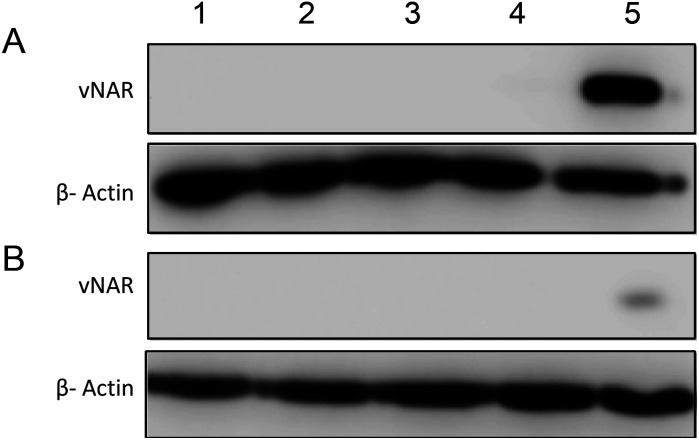
Delivery of vNAR into cells using cationic nanoparticle delivery system. HeLa cells were seeded and left overnight to adhere. Cells were treated with either vNAR PLGA or vNAR 10% PEI nanoparticles along with relevant controls for both (A) 3 and (B) 6 h timepoints. (1) Untreated, (2), PLGA NP, (3) 10% PEI NP, (4) vNAR PLGA NP, (5) vNAR 10% PEI NP. Post treatment, cells were washed with both PBS and an acid strip wash to remove any nanoparticles yet to be internalised before collecting cells for assessment *via* western blot. Lysates were collected, with cells incubated with his-NTA beads to select those cells containing the x6-his tag present on the C terminal of the vNAR. Samples were centrifuged to pull down any intact nanoparticles or polymer, ensuring the isolated supernatant contained only the soluble fraction representing what is inside the cell. Soluble fraction of supernatant was separated *via* SDS-PAGE and western blotting. Protein was detected using an anti-x6-his antibody, where band at ∼11 kDa is the approximate weight of vNARs. β-Actin was used as a protein loading control where a band is visible at ∼42 kDa. Data representative of three independent experiments.

Moreover, there was no apparent localisation of vNAR PLGA nanoparticles within any endo–lysosomal compartment. The degree of colocalisation was quantified *via* Pearson's correlation coefficient (*R*) using ImageJ colocalisation analysis, where *R* +1 indicated perfect association and *R* < 0 indicated no correlation. Pearson's correlation values indicated there was moderate correlation in colocalisation of staining with vNAR 10% PEI-PLGA nanoparticles and EEA1 (*R* = 0.2), whereas there was no association with LAMP1 (*R* = −0.42). After demonstrating enhanced uptake, next we assessed whether we could detect the nanoparticle cargo within the cell after treatment. HeLa cells were treated for 3 h and 6 h timepoints with PLGA and 10% PEI-PLGA nanoparticles containing vNAR which contains a C-terminal his tag. Following treatment cells were collected and upon lysis, each sample was incubated with Ni-NTA IMAC resin to allow capture of the HIS-tagged vNAR before assessment by western blotting. Upon blotting against the x6-his tag, a distinctive band is observed (∼11 kDa, the approximate weight of the x6-his tagged vNAR) in lane 5, indicative of those cells treated with the cationic 10% PEI-PLGA nanoparticle, where a larger band is observed after 3 h treatment compared to 6 h (Fig. 11A and B).

This is suggestive that the vNAR remains intact once delivered intracellularly, where more protein is observed 3 h post-treatment indicating that this is the optimal timepoint for detection, where after 6 h, it is possible that the his tag may be subject to proteolytic cleavage. These findings correlate with previous data presented, where the vNAR 10% PEI-PLGA nanoparticle demonstrated the ability for enhanced uptake into the cell.

## Discussion

Despite the identification of promising intracellular targets within a range of disease states, the progression of associated therapeutics to the clinic has been underwhelming. Attempts to date have been limited by the inefficiency of small molecules in binding targets of interest, whilst more promising biologics have been restricted by stability issues, and sub-optimal delivery mechanisms. Here we report a PEI nanoparticle delivery system which is not only capable of endo–lysosomal escape to achieve intracellular delivery of a vNAR cargo, but also has limited cellular toxicity. Nanoparticles were characterised by assessment of nanoparticle diameter, monodispersity and zeta potential. Nanoparticle size and PDI can affect particle biodistribution, as any fluctuation in particle diameter can alter both biodistribution and bioavailability, with the ideal size of nanoparticles for endocytosis and tumour filtration has been said to range between 100–300 nm.^33,36–40^

Furthermore, cationic nanoparticles have been shown to accelerate the rate of nanoparticle uptake as well as facilitate endo/lysosomal escape facilitating delivery of nanoparticle cargo to the cytosol.^30,41,42^ Physical characterisation of particles indicated that the 10% PEI-PLGA nanoformulation possessed the optimal characteristics for delivery, whilst also remaining stable for a period of up to 3 weeks. Whilst this nanoparticle formulation has been shown to be stable for storage at a range of conditions for this time period, stability is a key component for drug formulation, thus once a final formulation has been established where a vNAR has been identified against a specific intracellular target/disease state, stability will need to be further assessed. Therefore, future work should include assessment of vNAR binding following nanoparticle release in a biological setting, where vNAR functionality can be assessed *in vitro* after an extended period of time.

In this work, a vNAR was chosen as the nanoparticle cargo. As mentioned above, vNARs provide many attractive properties which are desirable when considering intracellular targets. vNARs are both the oldest (420 million years) and smallest (∼11 kDa) single chain domains acknowledged within vertebrates^17,43^ and exhibit many distinctive properties attributed to their unique origin within the adaptive immune system of sharks, such as significant stability and high affinity for their target, desirable features for intracellular delivery.^17,44^ Within their structure, the presence of a fourth binding loop and protruding paratopes facilitated by non-canonical disulfide bonds enables a distinctive ‘canyon binder’ targeting approach. The unique structure of vNARs also facilitates their propensity to bind unique and cryptic epitopes, inaccessible to more conventional biologics.^16^ Aside from their remarkable specificity and affinity for their target antigen, vNARs are subject to exposure to harsh conditions within their natural shark sera environment, which is characterized by having high levels of salt and urea. This has resulted in a molecule which possesses considerable stability, making them ideal for the harsh conditions which are characteristic of the intracellular environment, such as extreme pH, as well as exposure to proteases, all whilst retaining their binding affinity.^16,17,45,46^ The remarkable stability associated with vNARs make them ideal candidates for nanoformulation, where it has been demonstrated that vNARs remain physically intact as well as retaining all binding activity after enduring the harsh sonication techniques and presence of solvents during the process of nanoparticle synthesis, however further work should consider the relationship with pH and charge on encapsulation and subsequent release.

Upon cellular internalisation, nanoparticles preferentially accumulate within the endo/lysosomal pathway owing to their size profile.^47,48^ Nanoparticles are internalised *via* endocytosis, where they fuse into the early endosome. This vesicle then matures into the late endosome and subsequently lysosome.^31^ Endosomal escape is essential for intracellular delivery of therapeutics, as failure of nanoparticles to escape the endosome leads to accumulation within the lysosome, where degradation of the entrapped therapeutic is probable.^30^ In this study we have demonstrated that our 10% PEI-PLGA nanoparticle delivery system not only enhances nanoparticle uptake, demonstrated by an 8-fold increase in nanoparticle uptake, but shown that nanoparticles are visibly present within the endo/lysosomal pathway *via* colocalisation studies. Furthermore, it is shown that 10% PEI-PLGA nanoparticles are able to effectively escape the endo–lysosomal pathway as a result of endo–lysosomal disruption, clearly demonstrated by calcein and acridine orange staining profiles. However, the precise mechanism of action has not been determined.

Numerous theories as to how nanoparticles can escape the endosome have been proposed, one being the ‘proton sponge’ effect.^28,49^ This phenomenon states that once incorporated within the acidic pH of the endosomal lumen, the amine groups present on the PEI branched chains become protonated, resulting in buffering of the endosomal pH. In turn, this creates an influx of chlorine ions and water resulting in rupture of the lysosomal membrane as a consequence of osmotic swelling. In addition, the repulsive forces between similarly charged amine groups leading to swelling of the cationic nanoparticle may also contribute to loss of membrane integrity of the endosome. As a result, the ‘proton sponge’ theory itself may account for this cellular toxicity.^49^ However, validity of the proton sponge effect as a mechanism of endosomal disruption has been questioned, where reports have shown no observational change in lysosomal pH after treatment with cationic polymers, therefore other potential mechanisms of endo/lysosomal escape have been proposed.^49^ Such methods include endosomal release as a consequence of direct interaction of the cationic nanoparticle with the endosomal membrane itself, resulting in membrane disruption, as well as the effect of nanoparticle polymer degradation resulting in increased osmotic pressure. In addition, mechanisms reported also include, membrane fusion, nanoparticle swelling and membrane destabilisation.^30,50^ Furthermore, the nanoparticle formulation could be further modified through the addition of pH sensitive polymers, to further tailor the nanoformulation to react to fluctuations in pH within the tumour microenvironment, where pH sensitivity can be further investigated.^51–55^

Whilst promising, utilisation of cationic particles has proven challenging. One of the key concerns regarding the potential clinical translation of such cationic nanoparticles is their increased cellular cytotoxicity, as demonstrated by studies which have shown that cationic nanoparticles at high concentrations may induce caspase 3/7 activation and PARP cleavage ultimately leading to cell death.^15^ Furthermore, internalisation of cationic nanoparticle formulations can often induce cell death due to their effect on various intracellular mechanisms such as lysosomal disruption and damage to the mitochondria.^35^ Cationic nanoparticles must have a sufficient positive charge to increase cellular uptake and subsequently escape the endosome; however, this must be balanced by the need to maintain biocompatibility and not induce significant cell death. Although cationic nanoparticles facilitate a greater rate of internalisation, it is essential that the nanoparticles must be well tolerated with negligeable risk to healthy tissues.^32,56–58^ Hence, in addition to stability, toxicity screening of cationic nanoparticles is key. Encouragingly, our preliminary studies indicate the cationic 10% PEI-PLGA nanoparticles show little to no toxicity as observed in a panel of cancer cell lines, with only minimal toxicity observed even after 72 h at the top polymer concentration.

## Conclusion

In summary, the application of a double emulsion and evaporation nanoparticle formulation technique successfully resulted in the formation of a cationic nanoparticle (∼250 nm) which can be successfully loaded with a protein cargo, in this case a vNAR. The vNAR was not only successfully entrapped within the nanoparticle core but was also shown to retain binding capacity following nanoparticle formulation and subsequent release. The incorporation of the cationic polymer (PEI) within the nanoformulation significantly improved nanoparticle uptake into cancer cells *in vitro*, as well as facilitating endosomal escape after nanoparticle endocytosis, all whilst causing minimal cytotoxicity. Future work should include assessment of vNAR functionality post-delivery against their target antigen as well as, examination into the exact mechanism of vNAR escape. In addition, further investigation of nanoparticle toxicity, biodistribution and pharmacokinetics should be assessed *in vivo*. Taken together, this study provides rationale and evidence for utilising PLGA-PEI nanoparticles for the delivery of biologics towards intracellular targets, whilst also highlighting the enormous untapped potential for using vNARs therapeutically within this space.

## Data availability

All data generated in this study are included within the main article and/or ESI† accompanying this paper.

## Conflicts of interest

The authors declare no conflict of interest.

## Supplementary Material

RA-013-D3RA06050K-s001
