# Supplementary material for: Development of a cationic polyethyleneimine-poly(lactic-co-glycolic acid) nanoparticle system for enhanced intracellular delivery of biologics
Source: RSC Adv. 2023 Nov 17;13(48):33721–35. doi: 10.1039/d3ra06050k (PMC10654694; doi:10.1039/d3ra06050k)
Supplement: RA-013-D3RA06050K-s001 [file RA-013-D3RA06050K-s001.pdf]

Electronic Supplementary Material (ESI) for RSC Advances.
This journal is © The Royal Society of Chemistry 2023

Supplementary Information

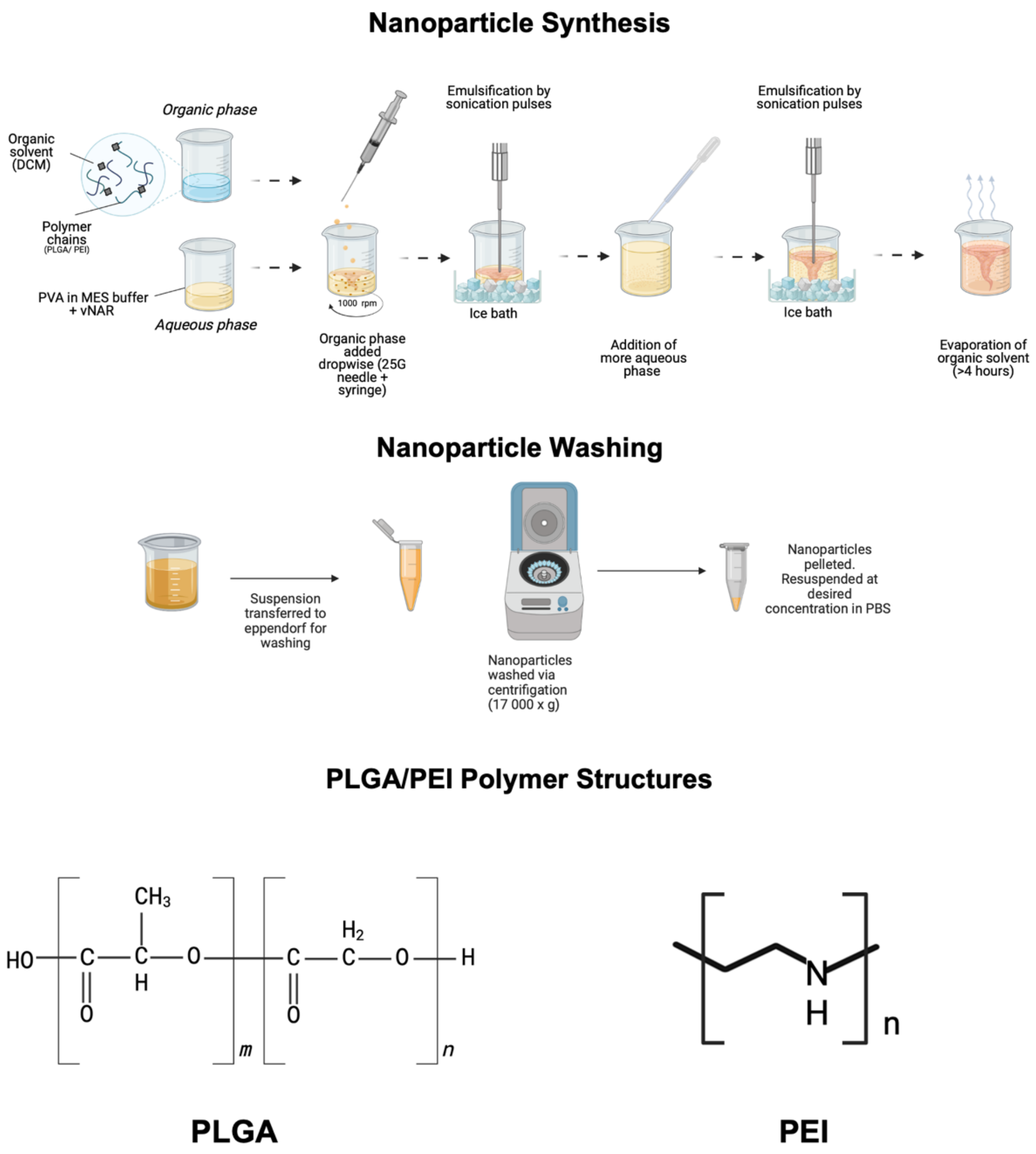

Supplemental 1 -Schematic depicting synthesis of PLGA-PEI Nanoparticles and PLGA/PEI polymer structures

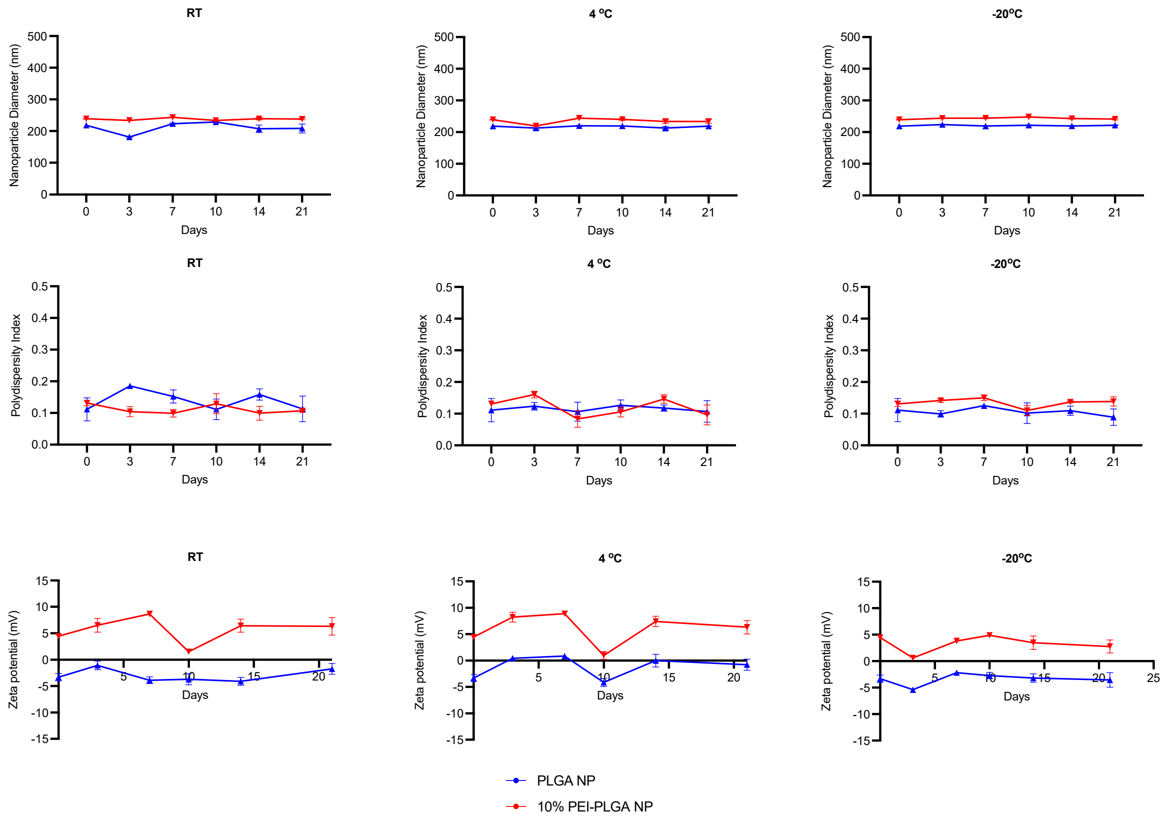

Supplemental 2: Assessment of nanoparticle stability

Nanoparticle diameter, polydispersity index zeta potential was measured for both PLGA and 10% PEI-PLGA Nanoparticles over a period of 21 days at various temperatures (RT, 4ºC, -20ºC) to assess NP stability.

A

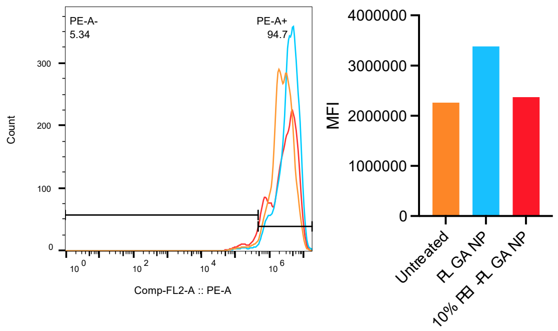

	Sample Name	MFI
	Untreated	2260000
	PLGA NP – 0.1 mg/mL	3380000
	10% PEI-PLGA NP – 0.1 mg/mL	2370000

B

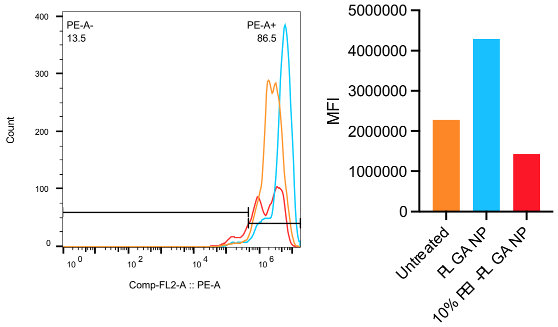

	Sample Name	MFI
	Untreated	2260000
	PLGA NP – 0.5 mg/mL	4290000
	10% PEI-PLGA NP – 0.5 mg/mL	1430000

C

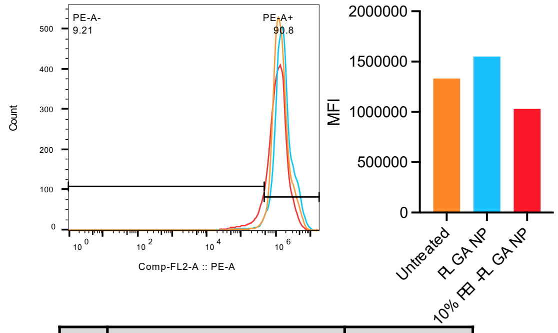

	Sample Name	MFI
	Untreated	1330000
	PLGA NP – 0.1 mg/mL	1550000
	10% PEI-PLGA NP – 0.1 mg/mL	1030000

D

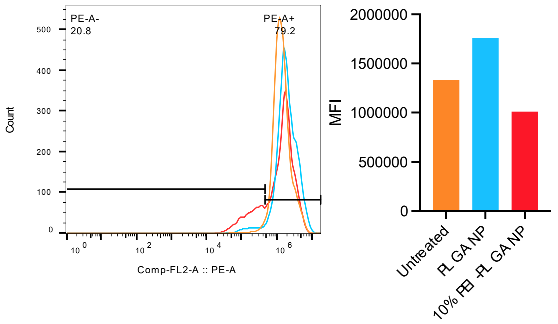

	Sample Name	MFI
	Untreated	1330000
	PLGA NP – 0.5 mg/mL	1760000
	10% PEI-PLGA NP – 0.5 mg/mL	1010000

Supplemental 3 - Cationic NP causes lysosome disruption

The ability of cationic nanoparticles to escape the lysosome was assessed via Acridine Orange staining. Hela cells were treated with 0.02- 0.5 mg/mL of PLGA or 10%-PEI-PLGA nanoparticles for (A-B) 24 h and (C-D) 48 h Following treatment cells were stained with acridine orange and cell fluorescence detected via FACS. Representative histograms show population shift relative to the untreated control for a low and high polymer concentrations of 0.1 mg/mL (A & C) and 0.5 mg/mL (B & D).where corresponding graphs show the mean fluorescence intensity (MFI). Data expressed as mean ± SEM (n=3). Significance was assessed by one-way ANOVA and Tukey's post-hoc test (**** $p<0.0001$, *** $p<0.001$, ** $p<0.01$, * $p<0.05$, ns $p>0.05$).

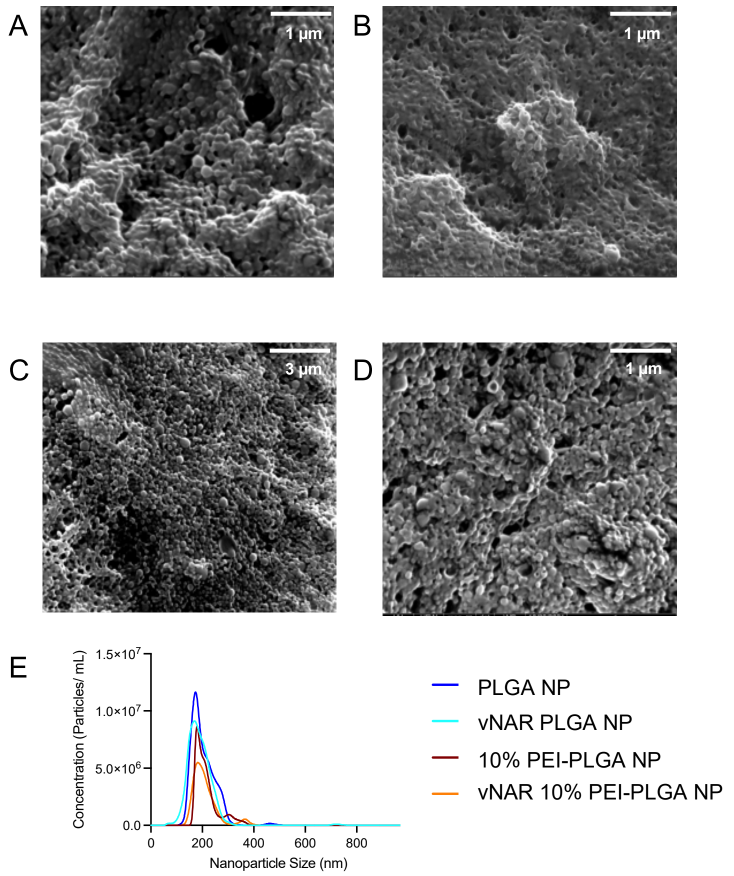

Supplemental 4: Nanoparticle Characterisation

Physicochemical properties of PLGA nanoparticles and 10% PEI-PLGA vNAR entrapped nanoparticles were further assessed via Electron Microscope (SEM) (A-D) and (E) nanoparticle tracking analysis (NTA).

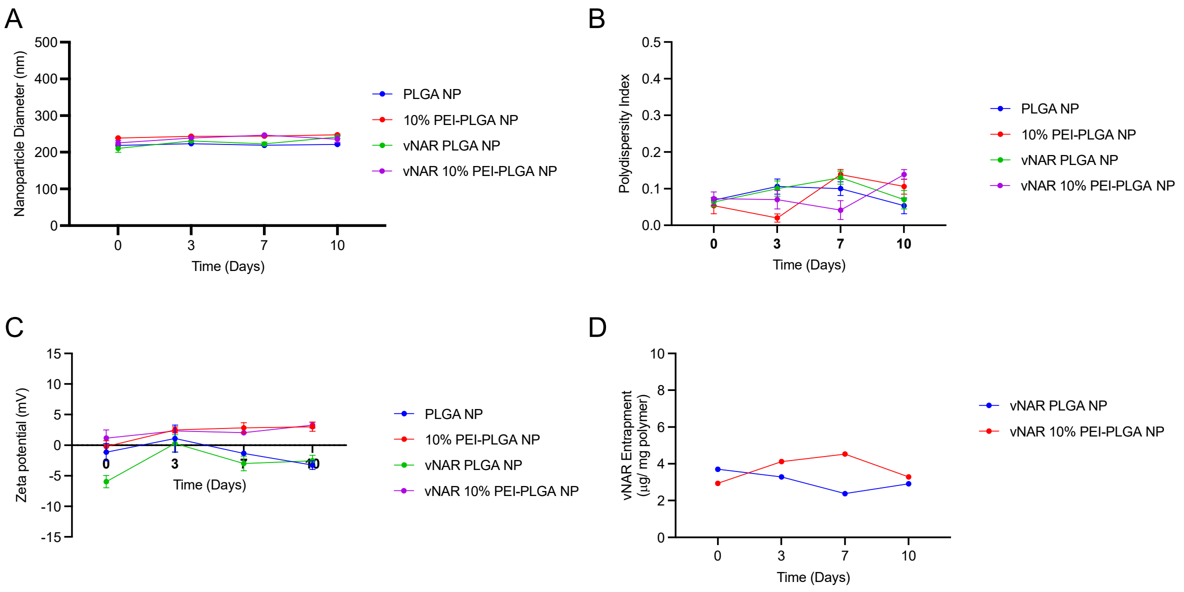

Supplemental 5: Assessment of cationic nanoparticle stability and vNAR loading

Nanoparticle was assessed in vNAR entrapped nanoparticle for a period of 10 days at 4°C where (A) nanoparticle diameter, (B) PDI, (C) zeta potential and (D) vNAR entrapment was quantified. Data expressed as mean ± SEM.
